# Supplementary material for: Predation and fragmentation portrayed in the statistical structure of prey time series
Source: BMC Ecol. 2009 May 6;9:10. doi: 10.1186/1472-6785-9-10 (PMC2689204; doi:10.1186/1472-6785-9-10)
Supplement: Additional file 2 — Voles and related classes ODDox Documentation. ODDox documentation of the agent-based model (ALMaSS) applied by Hendrichsen et al. The documentation is started by activating main.html. [file 1472-6785-9-10-S2.zip › Vole_ODDox/_predators_8cpp.html]

ALMaSS ODDox: Predators.cpp File Reference

- Main Page
- Related Pages
- Classes
- Files

# Predators.cpp File Reference

---

## Detailed Description

**The main source code for all predator lifestage and population manager classes**

Version of 28 January 2001.   
By Chris J. Topping   
  

`#include "Predators.h"`  
`#include "VolePopulationManager.h"`  

|  |  |
| --- | --- |
|  |  |
| Defines | |
| #define | \_CRT\_SECURE\_NO\_DEPRECATE |
| #define | OWL   1 |
| #define | WEASEL   0 |
| Functions | |
| static CfgInt | cfg\_owl\_breed\_day ("OWL\_BD", CFG\_CUSTOM, 120) |
| static CfgInt | cfg\_owl\_breed\_threshold ("OWL\_BT", CFG\_CUSTOM, 5000000) |
| static CfgInt | cfg\_owl\_death\_threshold ("OWL\_DT", CFG\_CUSTOM,-1) |
| static CfgInt | cfg\_owl\_StartingNo ("OWL\_START\_NO", CFG\_CUSTOM, 0) |
| static CfgInt | cfg\_weasel\_breed\_day ("WEASEL\_BD", CFG\_CUSTOM, 115) |
| static CfgInt | cfg\_weasel\_breed\_threshold ("WEASEL\_BT", CFG\_CUSTOM, 5) |
| static CfgInt | cfg\_weasel\_death\_threshold ("WEASEL\_DT", CFG\_CUSTOM, 10) |
| static CfgInt | cfg\_weasel\_DispersalMax ("WEASEL\_DISPERSAL\_MAX", CFG\_CUSTOM, 200) |
| static CfgInt | cfg\_weasel\_home\_range ("WEASEL\_HOME\_RANGE", CFG\_CUSTOM, 250) |
| static CfgInt | cfg\_weasel\_kill\_efficiency ("WEASEL\_KILL\_EFF", CFG\_CUSTOM, 250) |
| static CfgInt | cfg\_weasel\_NoFailuresBeforeDispersal ("WEASEL\_FAILURES", CFG\_CUSTOM, 10) |
| static CfgInt | cfg\_weasel\_search\_area ("WEASEL\_SEARCH\_AREA", CFG\_CUSTOM, 100) |
| static CfgInt | cfg\_weasel\_StartingNo ("WEASEL\_START\_NO", CFG\_CUSTOM, 0) |
| Variables | |
| int | owl\_breed\_day |
| int | owl\_breed\_threshold |
| int | owl\_death\_threshold |
| int | owl\_StartingNo |
| int | weasel\_breed\_day |
| int | weasel\_breed\_threshold |
| int | weasel\_death\_threshold |
| int | weasel\_StartingNo |

---

## Define Documentation

#define \_CRT\_SECURE\_NO\_DEPRECATE

#define OWL   1

Referenced by Owl::BeginStep(), TPredator\_Population\_Manager::CreateObjects(), and TPredator\_Population\_Manager::TPredator\_Population\_Manager().

#define WEASEL   0

Referenced by Weasel::BeginStep(), TPredator\_Population\_Manager::CreateObjects(), and TPredator\_Population\_Manager::TPredator\_Population\_Manager().

---

## Function Documentation

|  |  |  |  |  |
| --- | --- | --- | --- | --- |
| static CfgInt cfg\_owl\_breed\_day | ( | "OWL\_BD" | , |  |
|  |  | CFG\_CUSTOM | , |  |
|  |  | 120 |  |  |
|  | ) |  |  | `[static]` |

Referenced by TPredator\_Population\_Manager::TPredator\_Population\_Manager().

|  |  |  |  |  |
| --- | --- | --- | --- | --- |
| static CfgInt cfg\_owl\_breed\_threshold | ( | "OWL\_BT" | , |  |
|  |  | CFG\_CUSTOM | , |  |
|  |  | 5000000 |  |  |
|  | ) |  |  | `[static]` |

Referenced by TPredator\_Population\_Manager::TPredator\_Population\_Manager().

|  |  |  |  |  |
| --- | --- | --- | --- | --- |
| static CfgInt cfg\_owl\_death\_threshold | ( | "OWL\_DT" | , |  |
|  |  | CFG\_CUSTOM | , |  |
|  |  | - | *1* |  |
|  | ) |  |  | `[static]` |

Referenced by TPredator\_Population\_Manager::TPredator\_Population\_Manager().

|  |  |  |  |  |
| --- | --- | --- | --- | --- |
| static CfgInt cfg\_owl\_StartingNo | ( | "OWL\_START\_NO" | , |  |
|  |  | CFG\_CUSTOM | , |  |
|  |  | 0 |  |  |
|  | ) |  |  | `[static]` |

Referenced by TPredator\_Population\_Manager::TPredator\_Population\_Manager().

|  |  |  |  |  |
| --- | --- | --- | --- | --- |
| static CfgInt cfg\_weasel\_breed\_day | ( | "WEASEL\_BD" | , |  |
|  |  | CFG\_CUSTOM | , |  |
|  |  | 115 |  |  |
|  | ) |  |  | `[static]` |

Referenced by TPredator\_Population\_Manager::TPredator\_Population\_Manager().

|  |  |  |  |  |
| --- | --- | --- | --- | --- |
| static CfgInt cfg\_weasel\_breed\_threshold | ( | "WEASEL\_BT" | , |  |
|  |  | CFG\_CUSTOM | , |  |
|  |  | 5 |  |  |
|  | ) |  |  | `[static]` |

Referenced by TPredator\_Population\_Manager::TPredator\_Population\_Manager().

|  |  |  |  |  |
| --- | --- | --- | --- | --- |
| static CfgInt cfg\_weasel\_death\_threshold | ( | "WEASEL\_DT" | , |  |
|  |  | CFG\_CUSTOM | , |  |
|  |  | 10 |  |  |
|  | ) |  |  | `[static]` |

Referenced by TPredator\_Population\_Manager::TPredator\_Population\_Manager().

|  |  |  |  |  |
| --- | --- | --- | --- | --- |
| static CfgInt cfg\_weasel\_DispersalMax | ( | "WEASEL\_DISPERSAL\_MAX" | , |  |
|  |  | CFG\_CUSTOM | , |  |
|  |  | 200 |  |  |
|  | ) |  |  | `[static]` |

Referenced by Weasel::Weasel().

|  |  |  |  |  |
| --- | --- | --- | --- | --- |
| static CfgInt cfg\_weasel\_home\_range | ( | "WEASEL\_HOME\_RANGE" | , |  |
|  |  | CFG\_CUSTOM | , |  |
|  |  | 250 |  |  |
|  | ) |  |  | `[static]` |

Referenced by Weasel::Weasel().

|  |  |  |  |  |
| --- | --- | --- | --- | --- |
| static CfgInt cfg\_weasel\_kill\_efficiency | ( | "WEASEL\_KILL\_EFF" | , |  |
|  |  | CFG\_CUSTOM | , |  |
|  |  | 250 |  |  |
|  | ) |  |  | `[static]` |

Referenced by Weasel::Weasel().

|  |  |  |  |  |
| --- | --- | --- | --- | --- |
| static CfgInt cfg\_weasel\_NoFailuresBeforeDispersal | ( | "WEASEL\_FAILURES" | , |  |
|  |  | CFG\_CUSTOM | , |  |
|  |  | 10 |  |  |
|  | ) |  |  | `[static]` |

Referenced by Weasel::Weasel().

|  |  |  |  |  |
| --- | --- | --- | --- | --- |
| static CfgInt cfg\_weasel\_search\_area | ( | "WEASEL\_SEARCH\_AREA" | , |  |
|  |  | CFG\_CUSTOM | , |  |
|  |  | 100 |  |  |
|  | ) |  |  | `[static]` |

Referenced by Weasel::Weasel().

|  |  |  |  |  |
| --- | --- | --- | --- | --- |
| static CfgInt cfg\_weasel\_StartingNo | ( | "WEASEL\_START\_NO" | , |  |
|  |  | CFG\_CUSTOM | , |  |
|  |  | 0 |  |  |
|  | ) |  |  | `[static]` |

Referenced by TPredator\_Population\_Manager::TPredator\_Population\_Manager().

---

## Variable Documentation

int owl\_breed\_day

Referenced by Owl::BeginStep(), and TPredator\_Population\_Manager::TPredator\_Population\_Manager().

int owl\_breed\_threshold

Referenced by Owl::BeginStep(), and TPredator\_Population\_Manager::TPredator\_Population\_Manager().

int owl\_death\_threshold

Referenced by Owl::BeginStep(), and TPredator\_Population\_Manager::TPredator\_Population\_Manager().

int owl\_StartingNo

Referenced by TPredator\_Population\_Manager::TPredator\_Population\_Manager().

int weasel\_breed\_day

Referenced by Weasel::BeginStep(), and TPredator\_Population\_Manager::TPredator\_Population\_Manager().

int weasel\_breed\_threshold

Referenced by Weasel::BeginStep(), and TPredator\_Population\_Manager::TPredator\_Population\_Manager().

int weasel\_death\_threshold

Referenced by Weasel::BeginStep(), and TPredator\_Population\_Manager::TPredator\_Population\_Manager().

int weasel\_StartingNo

Referenced by TPredator\_Population\_Manager::TPredator\_Population\_Manager().

---

Generated on Thu Jan 22 14:13:45 2009 for ALMaSS ODDox by 
 1.5.6
